# PdAg/Ag(111) Surface Alloys: A Highly Efficient Catalyst of Oxygen Reduction Reaction

**DOI:** 10.3390/nano12111802

**Published:** 2022-05-25

**Authors:** Minghao Hua, Xuelei Tian, Shuo Li, Xiaohang Lin

**Affiliations:** Key Laboratory for Liquid-Solid Structural Evolution and Processing of Materials, Ministry of Education, School of Materials Science and Engineering, Shandong University, Jinan 250061, China; huaminghao@outlook.com (M.H.); 18366111497@163.com (S.L.)

**Keywords:** oxygen reduction reaction, DFT calculation, PdAg/Ag(111) surface alloy, single-atom alloy, efficient catalyst

## Abstract

In this article, the behavior of various Pd ensembles on the PdAg(111) surfaces was systematically investigated for oxygen reduction reaction (ORR) intermediates using density functional theory (DFT) simulation. The Pd monomer on the PdAg(111) surface (with a Pd subsurface layer) has the best predicted performance, with a higher limiting potential (0.82 V) than Pt(111) (0.80 V). It could be explained by the subsurface coordination, which was also proven by the analysis of electronic properties. In this case, it is necessary to consider the influence of the near-surface layers when modeling the single-atom alloy (SAA) catalyst processes. Another important advantage of PdAg SAA is that atomic-dispersed Pd as adsorption sites can significantly improve the resistance to CO poisoning. Furthermore, by adjusting the Pd ensembles on the catalyst surface, an exciting ORR catalyst combination with predicted activity and high tolerance to CO poisoning can be designed.

## 1. Introduction

The rapid development of human society increases the demand for energy, which would then further exacerbate the environmental problems, such as climate change and air pollution, caused by the consumption of non-renewable fossil fuels. Therefore, there is an urgent need to develop clean, renewable, and high-capacity energy conversion/storage technologies [1,2], such as the use of fuel cells, which is an efficient and promising energy conversion technology with clean reaction products and zero greenhouse gas emissions [3]. However, the broad deployment of fuel cells is still a challenge due to the high cost and sluggish oxygen reduction reactions (ORR) of cathode electrocatalysts [4,5]. Despite that great efforts have been made to search for new ORR catalysts, the most effective catalysts currently used for ORR are still pure platinum (Pt) and its alloys [6,7]. Many noble metal-based catalysts, especially Pt-based electrocatalysts, are susceptible to inactivity due to carbon monoxide (CO) poisoning. CO molecules block the reaction pathway by binding tightly to the active site, thus limiting the overall efficiency of proton exchange membrane (PEM) devices. In this case, the development of highly active, durable, and low-cost non-Pt catalysts resistant to CO has attracted great interest [8,9,10].

To reach a feasible stage of practical and general use of PEM fuel cells, one of the main obstacles is to develop a new catalyst at least as active as Pt-based metals, but with a lower noble metal content. The relative composition and distribution of metallic substances in bimetallic structures change as a result of the simultaneous presence of various competing effects, i.e., electron ligand, strain, and ensemble effects [11]. Non-linear changes in reactivity and selectivity are usually observed. In this case, it becomes important to understand these various effects [12]. A promising strategy is to dope non-reactive host materials with trace amounts of transition metal atoms as active centers, in order to form the monomer of transition metal on the surface, the so-called single-atom alloy (SAA) [13,14]. Generally speaking, the unique geometric properties of these SAA catalysts have improved the selectivity and stability of hydrogen-related reactions, C–C coupling, oxygen reduction, and CO_2_ reduction, as well as higher tolerance to catalytic poisoning [10,15,16,17].

Currently, palladium (Pd) is one of the most active transition metals with ORR activity comparable to Pt in alkaline media, which exhibits similar catalytic behavior and long-term durability [18,19,20]. A further advantage of Pd and its alloys is that it is considerably cheaper and has higher CO tolerance [21,22]. Alloying Pd with cheaper non-reactive host metals has been reported to be an effective strategy to improve its ORR activity and make PEM more economical [23,24,25]. For the ORR in alkaline media, silver (Ag) shows potential as a candidate to be used in bimetallic alloys with Pd due to its less-expensive cost and base stability [26,27,28]. Although Ag is approximately ten times less active than Pt atoms, previous experimental studies have shown that Ag-based bimetallic alloys have enhancement in the ORR behavior [29,30,31]. Recently, the behavior of PdAg surface alloys has been intensively investigated by experiments and theoretical calculations, especially in the reactive gas conditions. For example, the composition of the near-surface region of a Pd_75%_Ag_25%_(100) single crystal during CO oxidation under oxygen-rich conditions using near-ambient pressure X-ray photoelectron spectroscopy (NAP-XPS) confirms that the amount of Pd in the surface region decreases with increasing temperature. CO causes Pd segregation into the topmost surface layer [32]. The strong effects of reactive gases such as CO or O_2_ on the alloy structure and composition were investigated by scanning tunneling microscope (STM) and Fourier transform infrared spectroscopy (FTIR) to illustrate the correlation of chemical properties with structural aspects of bimetallic surfaces of a given composition at ambient pressure [33,34,35]. Theoretical calculations show that the driving force for the structural reconstruction is the strong interaction of the surface Pd sites with the adsorbed CO molecules, which changes the surface energy and leads to surface segregation of Pd [36,37]. Therefore, the surface structure can be intentionally adjusted by specific treatment of the catalyst in a special gas atmosphere. What is worthy of our attention is the theoretical studies on the mechanism of the oxygen reduction reaction in pure metals or bimetallic alloys under alkaline conditions, aiming to understand the detailed thermochemical processes of the active site’s pair of adsorbed hydroxide reactants and intermediates [38,39,40,41,42,43]. It is also proposed that predictors of catalyst activity can use the oxygen binding energy as a descriptor [44,45]. The catalyst activity prediction based on this descriptor has been shown to be in good agreement with experimental results [46]. Understanding the enhanced mechanisms behind the surface structure of alloy-based catalysts can guide the discovery of optimal reactivity/selectivity/stability and obtain promising catalysts for more complex systems. In this study, we will explore the possibility of using site-specific local reactive modifications to break the usual scaling relationships when designing new bimetallic catalysts for ORR. We follow the correlation mechanism of ORR on fcc metal (111) surfaces in earlier works, where the ORR pathway involves four proton–electron transfers and the reaction intermediates hydroperoxyl (OOH), oxygen (O), and hydroxide (OH) [40,45]. We calculate the adsorbate binding energies to determine their catalytic properties using the simple model suggested by Nørskov et al. [47]. Details of the associative reaction pathway and DFT calculations are provided in the Method Section.

According to our previous simulation and experimental results [48], using a combined casting and quenching strategy, PdAg alloy shows an obvious component segregation corresponding to the depth from the surface. A significant amount of Ag is observed on the first layer. The Pd atoms tend to disperse and form small clusters (monomer, dimer, and trimer), which shows good agreement with the experimental evidence. The main aim of this study is to investigate the catalytic activity of the special structures found in our previous research. The Pd(Ag)(111) is the close-packed surface of the face-centered cubic (fcc) crystal, and the (111) surface has the largest exposed area of Wulff shape [49]. As an example of small Pd ensembles (such as monomers and dimers), a snapshot of the first-layer structure of PdAg(111) with 50% Pd at 1200 K was shown in Figure S1 in the Supplementary Materials. Compared to the SAA surface annealed by magnetron sputtering, this strategy also produces a second layer of Pd enrichment on the alloy surface. Obviously, the PdAg SAA alloy prepared by the casting and quenching strategy is a potential candidate as a new catalyst of fuel cells, and this unique distribution might play a key role in the catalytic properties.

In this paper, a density functional theory (DFT) study on the reactivity and thermodynamics effects of Pd ensembles on the PdAg(111) surface is presented to assess the effect of different sizes and configurations on the ORR activity. To account for effects introduced by subsurface Pd, we also investigated PdAg(111) surface alloys with a Pd subsurface layer underneath. These different surface structures with ORR intermediates (OOH, O, and OH) were considered using the limiting potential as a metric of activity to study their trends, as influenced by the size of the Pd ensembles. Finally, we used the adsorption energy of CO to assess the extent of CO poisoning on the surface of these alloys compared with Pt(111).

## 2. Method

### 2.1. Density Functional Theory Calculations

All the DFT calculations in this study were performed using the Vienna Ab-initio Simulation Package (VASP) [50,51]. The projector-augmented wave (PAW) method was applied to treat electron–ion interactions, and the Perdew–Burke–Ernzerhof (PBE) exchange-correlation functional within the generalized gradient approximation (GGA) was employed to describe the electron interactions with a cutoff energy of 500 eV [52,53]. All structures were fully relaxed until the energy and force reached the convergence thresholds of 10^−5^ eV and 0.01 eV/Å. For the first Brillouin zone integration, the 25 × 25 × 25 and 5 × 5 × 1 Γ-centered *k*-point meshes were used at structure optimization for the bulk unit cell and slab models, respectively. For the calculation of the electronic structure of the surfaces, a k-point of 10 × 10 × 1 was used. The convergence of the results with respect to all the above parameters was carefully checked.

**Figure 1:**
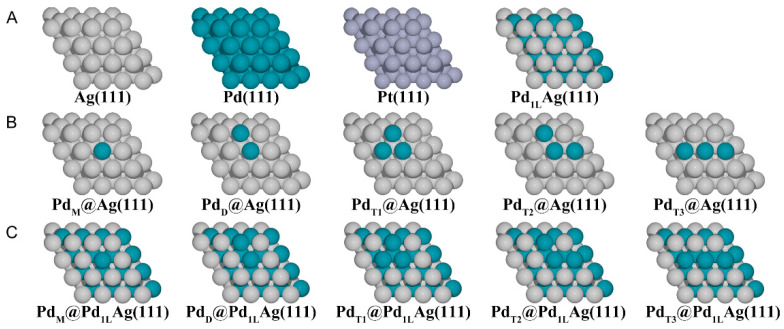
Considered geometries in this work. (**A**) The pure metal surfaces, Ag(111), Pd(111), and Pt(111), (**B**) modified PdAg(111) surfaces on the first layer, (**C**) modified PdAg(111) surfaces with a Pd subsurface layer. Pd monomer (indicated as M), dimer (D), and trimer (T). The silver, cyan, and gray balls represent Ag, Pd, and Pt atoms, respectively.

The simulated bulk lattice parameters obtained for Pd, Pt, and Ag were 3.94 Å, 3.97 Å and 4.15 Å, respectively, which are in good agreement with the experimental values of 3.89 Å (error: 1.3%), 3.92 Å (error: 1.3%), and 4.09 Å (error: 1.4%) [41,54]. The slabs were separated by 20 Å of vacuum in the perpendicular z-direction to avoid interactions between the two periodic units, and a dipole correction was applied. The surfaces were modeled by a 5-layer (4 × 4) supercell. The bottom three layers of each slab were fixed with the bulk lattice constant of the corresponding host metal (Ag, 4.15 Å), while the top two layers and the adsorbed species were fully relaxed. A detailed illustration of the considered structures is shown in Figure 1 for the various configurations. For the various structures studied in this work, we have used the following notation: Pd_1L_Ag(111) denotes a surface alloy at the topmost layer and a Pd sublayer underneath. In the case of a surface alloy, a certain number of Ag atoms (1–3 Ag atoms) at the topmost Ag overlayer of a Pd_1L_Ag(111) structure were replaced by Pd atoms. Note that, in our model (Figure 1C), the whole second layer was completely replaced by Pd atoms to simulate the fact that Pd is highly enriched in the second layer [48]. The pure metal surfaces (Ag(111), Pd(111), and Pt(111) in Figure 1A) and modified Ag(111) surfaces that were replaced by Pd atoms only on the first layer (Figure 1B) were also investigated to compare with the Pd_1L_Ag(111) structures. The formation energies of Pd ensembles (monomer (M), dimer (D), and trimer (T)) in these structures were calculated to investigate their relative stability. Here, the ensemble formation energy per Pd atom is given by:

(1) $$E_{f}=\left[E_{PdAg}-E_{Ag\left(111\right)}+N_{Pd}\left(E_{Ag-bulk}-E_{Pd-bulk}\right)\right]/N_{Pd}$$

where $E_{PdAg}$, $E_{Ag\left(111\right)}$, $E_{Ag-bulk}$, and $E_{Pd-bulk}$ represent the total energies of Pd_X_@Ag(111) or Pd_X_@Pd_1L_Ag(111) (X = M, D, T1, T2, T3), pure Ag(111), bulk Ag (per atom), and bulk Pd (per atom), respectively, and $N_{Pd}$ indicates the number of Pd atoms in the calculated surfaces.

To identify the energetically most favorable configuration, various possible adsorption sites were considered, including the top (T), bridge (B), and hollow (H) sites for the adsorbates (i.e., O, OH, OH, and CO). All geometric structures were visualized by using the Atomic Simulation Environment (ASE) [55]. The charge density distribution was visualized using VESTA [56]. The Bader charges were calculated using the Bader Charge Analysis Code [57].

### 2.2. Oxygen Reduction Reaction

The ORR pathway usually involves four electron transfers and at least three intermediates [46,58]. The associative reaction pathway is given by:

(2) $$O_{2}+*+\left(H^{+}+e^{-}\right)\to *OOH$$

(3) $$*OOH+\left(H^{+}+e^{-}\right)\to *O+H_{2}O$$

(4) $$*O+\left(H^{+}+e^{-}\right)\to *OH$$

(5) $$*OH+\left(H^{+}+e^{-}\right)\to *+H_{2}O$$

where * denotes the adsorption site for oxygen-containing intermediates.

The computational hydrogen electrode (CHE) model was adopted to calculate the adsorption-free energies of the four-electron transfer step for ORR, which defined the chemical potential of a proton–electron pair ($H^{+}+e^{-}$) equal to half of the chemical potential of gaseous $H_{2}$ at pH=0 in the electrolyte, 1 bar of $H_{2}$ in the gas at 298.15 K, and 0 $U_{RHE}$. (where RHE is the reversible hydrogen electrode) [59]. To avoid the use of $O_{2}$. electronic energy, which is difficult to determine accurately within standard GGA-DFT [60], we reference the experimental formation energy of $H_{2}O$ (4.92 eV) and DFT-calculated energies of $H_{2}O$. and $H_{2}$ molecules to deduce the binding energy of each intermediate, which was used as follows:

(6) $$2H_{2}O\to O_{2}+2H_{2},\;\Delta G=4.92\;\mathrm{eV}$$

The reaction-free energy of each elementary step was calculated as follows:

(7) $$\Delta G=\Delta E+\Delta ZPE+\Delta \int^{\;}C_{P}dT-T\Delta S$$

where ΔE is the difference in DFT-calculated electronic energy, ΔZPE is the zero-point energy (ZPE) change, $\Delta \int^{\;}C_{P}dT$ is the enthalpy change, T is the absolute temperature (here, T = 298.15 K), and ΔS is the entropy change for the reaction. All thermodynamic data were processed with VASPKIT code [61]. The adsorption-free energies were calculated as follows:

(8) $$\Delta G_{*OOH}=G_{*OOH}-G_{*}-G_{OOH}$$

(9) $$\Delta G_{*O}=G_{*O}-G_{*}-G_{O}$$

(10) $$\Delta G_{*OH}=G_{*OH}-G_{*}-G_{OH}$$

where $G_{*}$, $G_{*OOH}$, $G_{*O}$, and $G_{*OH}$ are the free energies of the clean substrate and the substrate binding with *OOH, *O, and *OH, respectively. $G_{OOH}$, $G_{O}$, and $G_{OH}$ are the free energies of isolated gas molecules, which can be replaced by $\left(2G_{H_{2}O}-\frac{3}{2}G_{H_{2}}\right)$, $\left(G_{H_{2}O}-G_{H_{2}}\right)$, and $\left(G_{H_{2}O}-\frac{1}{2}G_{H_{2}}\right)$, respectively. $G_{H_{2}O}$ and $G_{H_{2}}$ are the energies of $H_{2}O$ and $H_{2}$ in the gas phase, where $H_{2}O\left(g\right)$ is considered at the vapor pressure of $H_{2}O\left(l\right)$ at 298.15 K.

The catalytic activities of ORR with different structures were estimated by determining the theoretical thermodynamic limiting potential with *OOH, *O, and *OH as reaction intermediates. The reaction-free energy of each elementary reaction step for electrochemical oxygen reduction can be defined as the difference between two adjacent steps, shown as follows:

(11) $$\Delta G_{1}=G_{*OOH}-G_{O_{2}}$$

(12) $$\Delta G_{2}=G_{*O}-G_{*OOH}$$

(13) $$\Delta G_{3}=G_{*OH}-G_{*OOH}$$

(14) $$\Delta G_{4}=G_{H_{2}O}-G_{*OH}$$

The theoretical thermodynamic ORR limiting potential ($U_{L}$) and overpotential (η), which can be the measure of the activity of a catalyst, are then defined from the largest ΔG among reactions (Equations (2)–(5)), as follows:

(15) $$U_{L}=-{\left\{\Delta G_{1}∕e,\Delta G_{2}∕e,\Delta G_{3}∕e,\Delta G_{4}∕e\right\}}_{\max}$$

(16) $$\eta =1.23\;V-U_{L}$$

For ORR, the theoretical minimum half-cell potential is 1.23 V. A higher $U_{L}$ value corresponds to a lower overpotential, η (1.23 V—$U_{L}$), indicating improved theoretical activity. The theoretical overpotential is able to be benchmarked by comparing it with that of Pt(111), which has an *η* of 0.43 V [62]. Hence, the activity of a novel catalyst is considered to be improved when it has η < 0.43 V ($U_{L}$ > 0.80 V). Note that η should not be compared directly with the experimentally measured overpotential, which depends on the current density [63]. Details of the adsorption energies, d-band centers, Bader charge, and atomic structures are provided in the Supplementary Materials.

## 3. Results and Discussion

### 3.1. Reaction Profile and Calculated Overpotentials

To clarify the stability of the constructed structures, the formation energies, $E_{f}$, of Pd ensembles (monomer, dimer, and trimers) were investigated as shown in Table 1. All formation energies were negative, indicating that it is thermodynamically possible to form these ensembles [8]. Similar formation energies of Pd monomers and dimers on Ag(111) (0.01 eV) and Pd_1L_Ag(111) (0.02 eV) suggest that the formation of Pd monomers and dimers should be almost equally favorable, which indicates that all kinds of ensembles could be distributed on the PdAg surfaces at high temperatures. Note that according to the number of Pd ensembles studied in previous MC simulations and experiments, the Pd monomers surrounded by Ag atoms at low temperatures are numerically more than the dimers and trimers. Therefore, the ORR overpotentials of Pd_M_@Ag(111) and Pd_M_@Pd_1L_Ag(111) have a greater influence on the catalytic performance [48].

According to the Sabatier principle, as an efficient catalyst, the binding between the active site and the reactants or the final products should not be too weak or too strong [40]. The reaction processes of the ORR intermediates on different surfaces were investigated and summarized. The efficiency of catalysts is often qualitatively gauged by overpotential (η) based on thermodynamics (lower η corresponds to greater predicted activity) [62]. Table 2 shows the overpotentials of all surfaces calculated using the formula in Equations (11)–(14). The reaction on Pd_M_@Pd_1L_Ag(111) had the lowest η (η = 0.41 V). Some surfaces had similar overpotential, such as Pd_D_@Ag(111) (η = 0.42 V), Pd_M_@Ag(111) (η = 0.46 V), and Pd_D_@Pd_1L_Ag(111) (η = 0.47 V), especially on Pd_M_@Pd_1L_Ag(111), where they even performed better. Due to the non-linear relationship between the adsorption energy and overpotential, as shown in Table S1, it is hard to quantitatively point out the influence of the Pd sublayer. However, it is obvious that the existence of the Pd sublayer significantly changes the activity of the surfaces. The lower overpotentials of the modified structures compared to pure Ag (0.62 V) in Table 2 are consistent with the realization that the surface contains Pd atoms surrounded by Ag atoms, as reported in the experimental literature, which maximizes the ability of the heteroatomic sites to amplify the activity of each Pd atom, thus enhancing the ORR activity [24,26].

**Figure 2:**
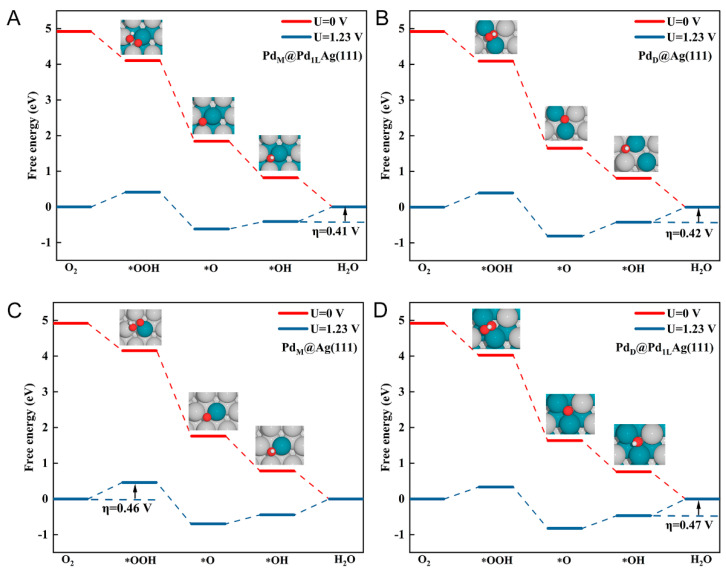
Gibbs free energy diagrams for the ORR pathway at the equilibrium potential (U = 1.23 V), and zero potential on the four lowest overpotential surfaces. (**A**) Pd_M_@Pd_1L_Ag(111), (**B**) Pd_D_@Ag(111), (**C**) Pd_M_@Ag(111), and (**D**) Pd_D_@Pd_1L_Ag(111). The silver, cyan, red, and white balls represent Ag, Pd, O, and H atoms, respectively.

To understand the reaction on the four best-performing surfaces in more depth, Figure 2 illustrates the ORR reaction profiles at various electrode potentials. It is obvious that all the reaction steps were downhill (negative free energy changes), implying a facile reaction at U = 0 V, and the ORR reaction intermediates can spontaneously adsorb on these structures. With the increasing electrode potential, the change of free energy became less negative. At the voltage U = 1.23 V, both the O and OH hydrogenation reactions became endothermic. Consequently, there is a highest electrode potential under which all reaction steps along the reaction decreased the free energy. This limiting potential defines the working potential of the electrocatalysts. Note that the potential-determining step (PDS) on the four best-performing surfaces lies in the reduction of O-containing species: OOH or OH. The PDS of ORR on the Pd_M_@Pd_1L_Ag(111), Pd_D_@Pd_1L_Ag(111), and Pd_D_@Ag(111) is located in the fourth elemental step (the reduction of OH to form the final production of H_2_O, Equation (5), and on the Pd_M_@Ag(111) surface is the reduction of the O_2_ to form OOH (Equation (2)).

For the surfaces Pd_M_@Ag(111), Pd_D_@Ag(111), and Pd_M_@Pd_1L_Ag(111), these two steps determined the same ORR potential, reflecting that both of them have a reactivity close to optimal ($\Delta G_{1}-\Delta G_{4}$ = −0.0155 eV for Pd_M_@Ag(111), $\Delta G_{1}$−$\Delta G_{4}$ = −0.0220 eV for Pd_D_@Ag(111), and $\Delta G_{1}$−$\Delta G_{4}$ = −0.0002 eV for Pd_M_@Pd_1L_Ag(111)). Generally, increasing ΔG for one of the steps will decrease ΔG for the other step due to the linear relations between the adsorption energies. Suitable reaction energy barriers lead to lower overpotential values and higher catalytic activity [64]. Interestingly, the adsorption of *OOH being rate-limiting for Pd_M_@Ag(111) reflects the fact that the surface binds the intermediates too weakly relative to the optimal ORR catalyst [40]. However, on the other hand, the lower overpotential of Pd_M_@Pd_1L_Ag(111) than that of Pd_M_@Ag(111) could also be explained by the fact that the introduction of the Pd sublayer enhances the adsorption of the intermediates.

**Figure 3:**
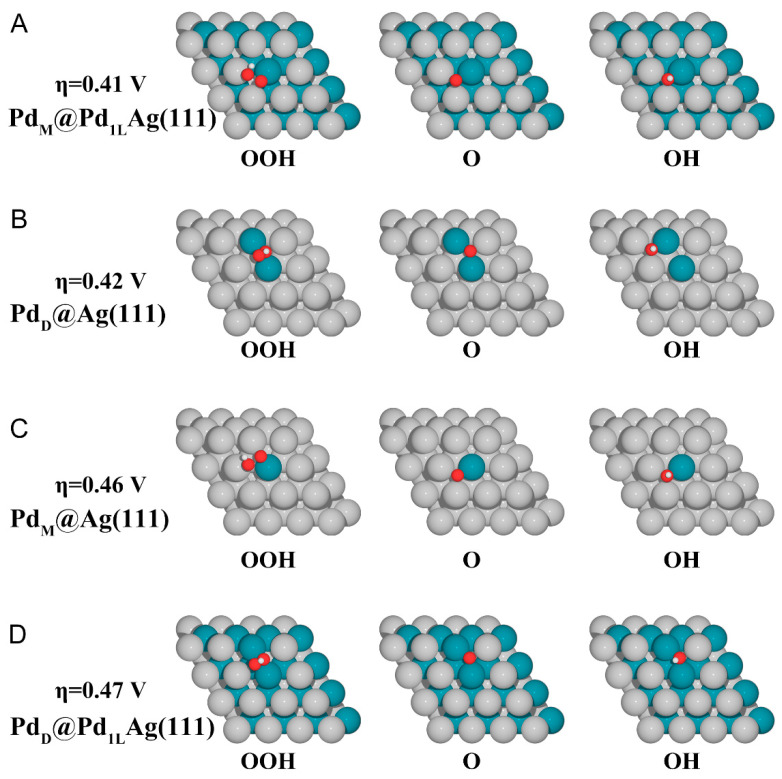
Top view of the atomistic structures of all ORR intermediates on the best four surfaces. (**A**) Pd_M_@Pd_1L_Ag(111), (**B**) Pd_D_@Ag(111), (**C**) Pd_M_@Ag(111), and (**D**) Pd_D_@Pd_1L_Ag(111). The silver, cyan, red, and white balls represent Ag, Pd, O, and H atoms, respectively.

Figure 3 shows details of the most favorable adsorption geometries of the ORR intermediates (*OOH, *O, and *OH) on the four best-performing surfaces (other surfaces are shown in Figure S2). For each intermediate, we examined its optimized adsorption site at various possible positions and then determined its lowest-energy adsorption conformation on the surface. In Table S1 and Figure S2, the calculated lowest binding energies and site preferences for various chemical species on the modified surfaces are listed. According to previous studies, the surface inhomogeneity and valence rules of adsorption sites of SAA thus exhibit unique catalytic properties, which can create new opportunities for designing catalysts with excellent performance [65]. As can be seen in Figure 3, the adsorption of intermediates mostly occurs at the bridge or hollow sites between the atoms of dissimilar metals. On SAA surfaces (Figure 3A,C), *OOH binds at the top-bridge site between the host (Ag) and dopant (Pd) atoms. The *OOH adsorbate is tilted and therefore nearly parallel to the surface. *OOH is bound to the surface through the interaction of the non-protonated O atom with the dopant Pd atom. The protonated O atom is bound to only one host atom close to the top site. For Pd dimer surfaces, *OOH prefers to adsorb in bridge sites and is tilted at a different angle than in the case of the Pd monomer surface.

For *O adsorbed on the surface, the only energetically most favorable conformation is at the fcc site. On all surfaces except the two T1-type surfaces, *O is bound at sites sharing the dopant–host atoms. For *OH, it binds to the hollow site. On the Ag(111)-based surface, *OH prefers the fcc site with one dopant atom and two host atoms, but on the Pd_1L_Ag surface, it prefers the fcc site with two dopant atoms and one host atom. Although we calculated different surface aggregates, it can be concluded that adsorption always occurs on landmark structures, such as monoatomic and dimeric aggregates for the adsorption process. Except for T1-type ensemble surfaces, *OH and *O absorbed on T2- and T3-type surfaces have the same behavior as the dimer and monomer because of similar structural features. The properties of the two T1-type Pd ensembles exhibit a pure Pd(111) behavior, which is also verified again later on the adsorption energy of CO. This decouples the binding strength of these fragments and thus defines a weakly correlated scaling relationship. The binding sites of these adsorbates vary depending on the size of the Pd ensembles, resulting in the decoupling of the adsorption strength.

**Figure 4:**
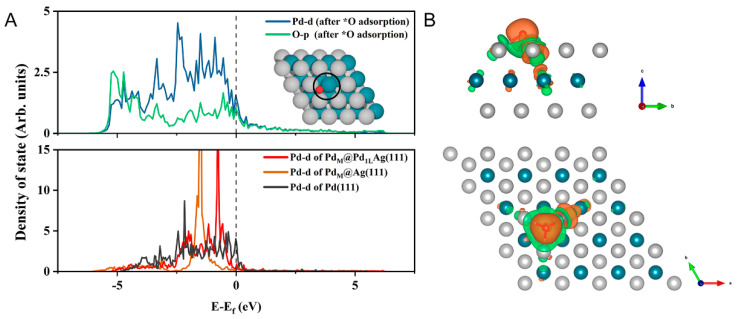
Electronic structure properties of Pd_M_@Pd_1L_Ag(111). (**A**) PDOS plot for the Pd dopant atom as well as the adsorbed O atom (top) and the selected Pd atom in Pd_M_@Pd_1L_Ag(111), Pd_M_@Ag(111), and Pd(111) (bottom). The position of the Fermi level is marked with the dashed line. (**B**) Isosurface of charge density distribution for adsorbed O atom on the Pd_M_@Pd_1L_Ag(111) system. The charge depletion and accumulation are depicted as cyan and orange colors, respectively. The isosurface value is 0.002 e/Bohr^3^. The silver, cyan, and red balls represent Ag, Pd, and O atoms, respectively.

To investigate the effect of the electronic structure on the ORR activity of the surface, partial density of states (PDOS) and charge density difference analysis of Pd_M_@Pd_1L_Ag(111) were performed, as shown in Figure 4. The d-projected density of states of Pd atoms on the Pd_M_@Pd_1L_Ag(111) and Pd_M_@Ag(111) surfaces was significantly different from that on the Pd(111) surface, which has a formation of a sharp peak near the Fermi level (lower part of Figure 4A). Compared with the pure Pd surface, an obvious d-DOS shift on bimetal surfaces was observed moving close to the Fermi level, which can contribute more electron density to the metal-adsorbate and thus improve the reactivity. The peak of Pd on Pd_M_@Pd_1L_Ag(111) was closer to the Fermi level than that of Pd_M_@Ag(111). After oxygen adsorption, the sharp peak of Pd on Pd_M_@Pd_1L_Ag(111) disappeared (upper part of Figure 4A). There was a strong hybridization and splitting in the bonding and anti-bonding states between the *O and Pd atoms at about −0.20 and 0.41 eV, which indicates a strong interaction between them. The high reactivity exhibited by the dopant Pd atom changes the site preference of the adsorbates so that they are adsorbed in close proximity to the Pd atom. PDOS with O atom adsorption showed surface–adsorbate interactions with contributions from the Pd d-band. The O atom can draw more electrons from the Pd atom on the first layer of the catalyst surface (Figure 4B, orange color indicates electron accumulation and blue indicates electron depletion). It can be seen that the Pd atom in the second layer lost electrons, and this electron transfer to the surface had more electron accumulation around the active center of the Pd atom, thus facilitating the electron transfer between O and the catalyst surface. The d-band center and Bader charge of the Pd atom in the slab models are summarized in Table S2. The d-band center varied from −1.617 to −1.292 eV, and the four best-performing surfaces had values of −1.545 eV (Pd_M_@Ag(111)), −1.496 eV (Pd_D_@Ag(111)), −1.329 eV (Pd_M_@Pd_1L_Ag(111)), and −1.292 eV (Pd_D_@Pd_1L_Ag(111)), respectively. Since the d-band center of these dopant Pd atoms is closer to the Fermi level compared to the same type of substrate surface (Ag(111) or Pd_1L_Ag(111)), it contributes more to the electron density of the mixed metal-adsorbent band than the lower d-band, thus improving the reactivity. Bader charge analysis also showed that the Pd atoms of the four surface models with high activity received more electrons from host metal atoms, which also corroborates the better activity.

**Figure 5:**
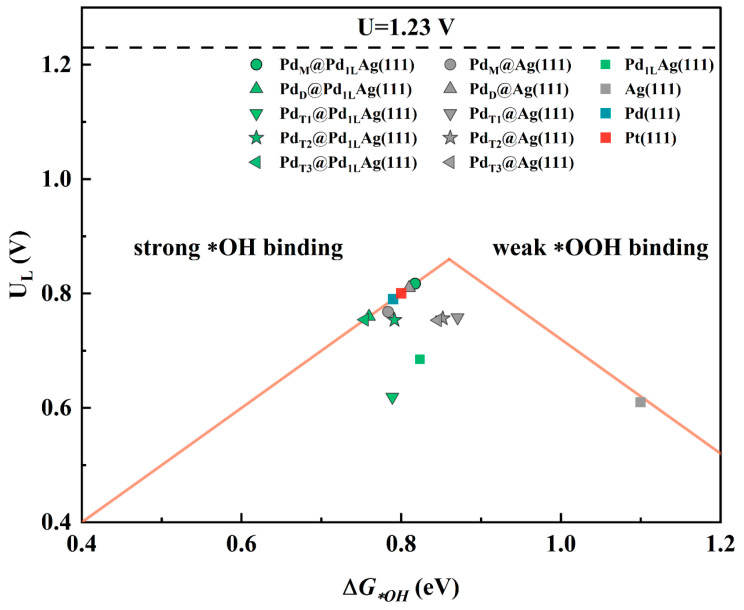
Volcano plot of the limiting potential for the ORR analyzed by results from the thermodynamic analysis. The black dashed line is the equilibrium potential for the ORR.

The ORR activity can be evaluated by the thermodynamic limiting potential, $U_{L}$, which depends on the surface binding energy of the reacting oxygen adsorbate. Thus, the relationship between $\Delta G_{*OH}$ and the limiting potential, $U_{L}$, of each fundamental process of ORR can be described by a volcano diagram, where the system at the peak is the best system. Based on the correlation between the binding energies of surface *OH and *OOH of different metals and their role in the thermodynamic limiting step of ORR, the orange theoretical volcano shown in Figure 5 was obtained. It can be clearly found that the limiting potential increased with $\Delta G_{*OH}$ and then decreased. When $\Delta G_{*OH}$ was smaller than 0.86 eV, on the left side of the volcano, the ORR was limited by the strong *OH adsorption at the metal center, which poisons the catalytic surface. When the *OH adsorption was weaker, in turn, the reaction behaved better, as shown in Figure 5. The species to the right of 0.86 eV on the *X*-axis illustrates the opposite situation, where the H_2_O molecule formed in the last hydrogenation step evolved into the RDS, i.e., the adsorption step was thermodynamically limited. In fact, only when $\Delta G_{*OH}$ was in a moderate range was the formation energy of the intermediate species neither too strong nor too weak, thus obtaining a relatively low overpotential [46]. As predicted, Pd_M_@Pd_1L_Ag(111) was shown to be closer to the peak of the volcano curve and can be the ideal active catalyst. In this case, the ORR can reach high catalytic performance. Unlike several existing experimental studies on the ORR reactions of PdAg clusters or thin-film alloys [24,62,66,67], here, we simulated a model of SAA with a special inner-layer structure which has been prepared by heat treatment combined with quenching in our previous experiments. As can be seen from Figure 5, all the structures had higher limiting potentials than pure Ag(111) ($U_{L}$ = 0.61 V), and Pd_M_@Pd_1L_Ag(111) and Pd_D_@Ag(111) were higher than pure Pd(111) ($U_{L}$ = 0.79 V), indicating a synergistic enhancement between Pd and Ag, which can provide solution ideas for the design of bimetallic catalysts. Note that the modified surface Pd_M_@Pd_1L_Ag(111) possesses limiting potentials beyond that of pure Pt(111) and is an excellent candidate for an ORR catalyst, where the influence of the inner layer plays an important role in the catalytic activity.

### 3.2. Reduced CO Poisoning of the Catalyst

**Figure 6:**
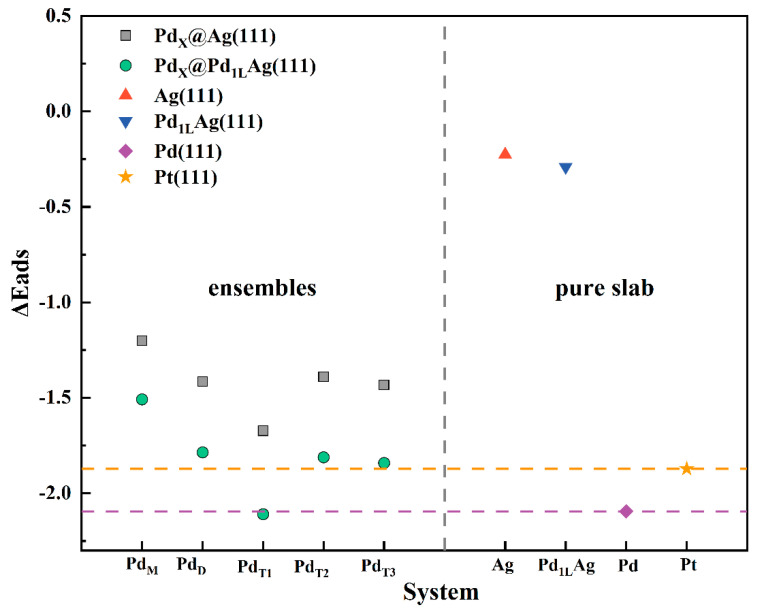
Adsorption energies of CO on different adsorption sites of the modified surfaces and pure slabs. The yellow dashed line is the adsorption energy of CO on pure Pt(111). The purple dashed line is the adsorption energy of CO on pure Pd(111).

**Figure 7:**
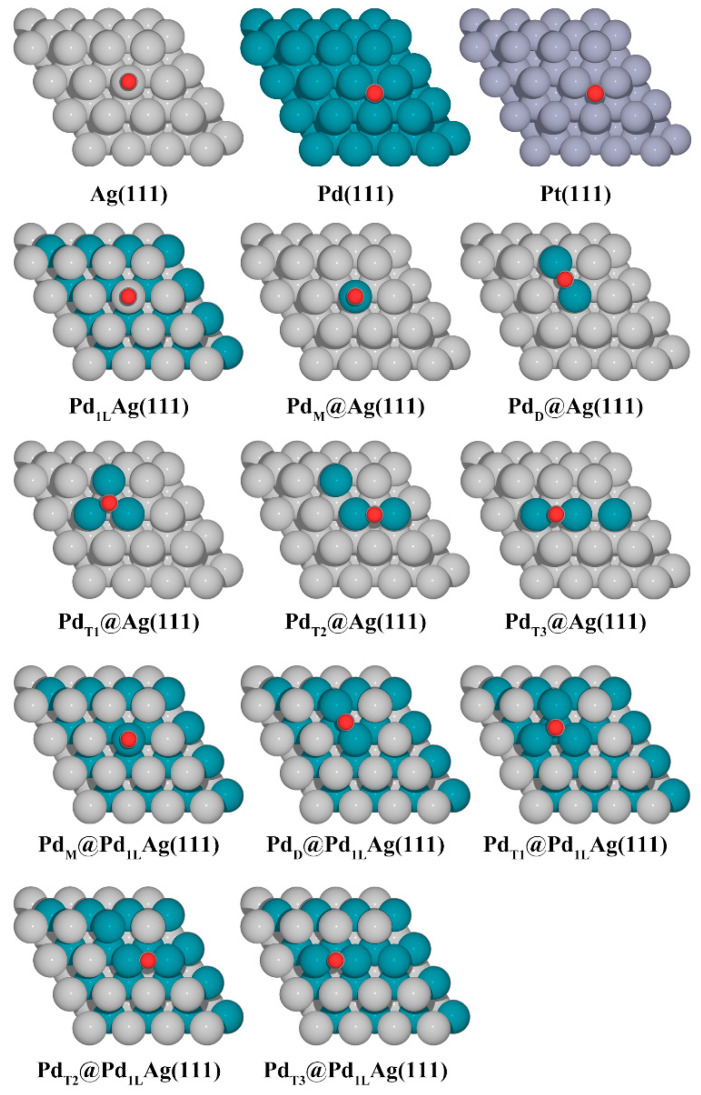
Top view of the most stable structures of CO on all slab models. The silver, cyan, gray, brown, and red balls represent Ag, Pd, Pt, C, and O atoms, respectively.

The above calculations indicate that the combination of Pd ensembles on the alloy surfaces designed and prepared in our previous work (especially Pd_M_@Pd_1L_Ag(111)) exhibits a theoretical overpotential comparable to that of Pt(111). Pt-based catalysts are easily poisoned by CO impurity gas even at low concentrations, resulting in decreased performance and a shortened service life. Most noble metal catalysts are susceptible to poisoning by CO molecules due to their inherent strong CO chemisorption, while Pt catalysts are highly susceptible to catalytic poisoning by CO. The adsorption energy of CO on each surface was calculated, as shown in Figure 6. The CO chemisorption on Pd_M_@Ag(111) was the weakest compared to pure Pd(111) and Pt(111) surfaces. It can be seen that the adsorption energies of all monomer and dimer structures were smaller than those of the pure Pt(111) surface, indicating that these structures are more tolerant to CO than the pure Pt(111) surface. Figure 7 shows that CO prefers to adsorb at the top site of the monomer and the bridge site of the dimer, while none of the intermediates of the ORR prefer to adsorb at these two sites, which would make the effect of CO weaker. The adsorption energies of the Pd_1L_Ag(111) surface were stronger than those of the Pd_X_@Ag(111) surfaces due to the substitution of the Ag atom in the second layer by Pd. The introduction of the Pd layer made the overall CO adsorption energies stronger by a fixed value, but still weaker than the adsorption energy on pure Pd(111) and pure Pt(111). The Pd trimer is the structure that determined the lowest adsorption energy. The adsorption energy of the Pd_T1_@Pd_1L_Ag(111) surface was close to that of pure Pd. Once the T1-type Pd trimers are available in the PdAg surface, they become the most favorable adsorption sites (on hollow sites). This ensemble effect is consistent with the experimental observation in [41]. Since the CO adsorption sites on the Pd monomer and dimer are at the top and bridge sites, respectively, the adsorption sites of the ORR intermediates on the monomer and dimer surfaces are *OOH at the top-bridge site (monomer) or bridge site (dimer), with *O and *OH at the hollow site. Therefore, the adsorption sites are not conflicting for the intermediates (except for the *OOH on the Pd dimer surfaces), which will lead to an increase in the catalysts’ surface resistance to poisoning.

## 4. Conclusions

In this article, we systematically investigated the behavior of various Pd ensembles on the PdAg(111) surfaces (with/without a Pd subsurface layer) for ORR intermediates. The most favorable adsorption conformations of *OOH, *O, and *OH on each structure were elucidated. A tendency of ORR intermediates to have different adsorption sites at different ensemble sizes was found. Furthermore, using the CHE model, the free energy profiles of whole ORR pathways were estimated and analyzed. The results showed that Pd_M_@Pd_1L_Ag(111) had the best predicted performance, with a higher limiting potential (0.82 V) than Pt(111) (0.80 V), which could be explained by the subsurface coordination. It could also be proven by the analysis of the electronic properties. In this case, it is necessary to consider the influence of the near-surface layers when modeling the SAA catalyst processes. Another important advantage of PdAg SAA is that atomic-dispersed Pd as adsorption sites can significantly improve the resistance to CO poisoning. The best surface in terms of resistance to CO poisoning was Pd_M_@Ag(111) with a single Pd atom. Furthermore, due to the wide variation of adsorption energy on these bimetallic surfaces, it is possible to precisely modulate the adsorption energy by adjusting the Pd surface content, combining activity with high tolerance to CO poisoning, which could constitute an exciting and ideal combination.

## Figures and Tables

**Table 1:** The ensemble formation energies, $E_{f}$, of all structures.

Active Site Model	$E_{f}\;(\mathrm{eV})$	Active Site Model	$E_{f}\;(\mathrm{eV})$
Pd_M_@Ag(111)	−0.12	Pd_M_@Pd_1L_Ag(111)	−0.08
Pd_D_@Ag(111)	−0.11	Pd_D_@Pd_1L_Ag(111)	−0.06
Pd_T1_@Ag(111)	−0.09	Pd_T1_@Pd_1L_Ag(111)	−0.04
Pd_T2_@Ag(111)	−0.10	Pd_T2_@Pd_1L_Ag(111)	−0.04
Pd_T3_@Ag(111)	−0.10	Pd_T3_@Pd_1L_Ag(111)	−0.04
		Pd_1L_Ag(111)	−0.10

**Table 2:** The reaction-free energies of the four e^−^ ORR reactions, as well as the corresponding theoretical limiting potential and overpotential.

Active Site Model	$\Delta G_{1}\left(eV\right)$	$\Delta G_{2}\left(eV\right)$	$\Delta G_{3}\left(eV\right)$	$\Delta G_{4}\left(eV\right)$	$U_{L}\left(V\right)$	η(V)
Pd_M_@Ag(111)	−0.768	−2.391	−0.977	−0.784	0.768	0.462
Pd_D_@Ag(111)	−0.832	−2.439	−0.840	−0.810	0.810	0.420
Pd_T1_@Ag(111)	−0.772	−2.520	−0.758	−0.871	0.758	0.472
Pd_T2_@Ag(111)	−0.756	−2.464	−0.847	−0.852	0.756	0.474
Pd_T3_@Ag(111)	−0.753	−2.490	−0.831	−0.846	0.753	0.477
Pd_M_@Pd_1L_Ag(111)	−0.817	−2.262	−1.023	−0.817	0.817	0.413
Pd_D_@Pd_1L_Ag(111)	−0.895	−2.390	−0.875	−0.760	0.760	0.470
Pd_T1_@Pd_1L_Ag(111)	−0.937	−2.575	−0.619	−0.789	0.619	0.611
Pd_T2_@Pd_1L_Ag(111)	−0.870	−2.505	−0.753	−0.792	0.753	0.477
Pd_T3_@Pd_1L_Ag(111)	−0.878	−2.420	−0.868	−0.754	0.754	0.476
Pd_1L_Ag(111)	−0.685	−2.129	−1.282	−0.824	0.685	0.545

## Data Availability

Not applicable.

## Supplementary Materials

The following supporting information can be downloaded at: https://www.mdpi.com/article/10.3390/nano12111802/s1, Table S1: The free energies of the adsorption of ORR intermediates in eV on all active site models; Table S2: d-band center and Bader charge of Pd atom at the first layer of all models; Figure S1: MC snapshots of the atomic structure of PdAg(111) with 50% Pd at 1200 K; Figure S2: Top view of the atomistic structures of all ORR intermediates on all considered surfaces.
