# Supplementary material for: PdAg/Ag(111) Surface Alloys: A Highly Efficient Catalyst of Oxygen Reduction Reaction
Source: Nanomaterials (Basel). 2022 May 25;12(11):1802. doi: 10.3390/nano12111802 (PMC9182455; doi:10.3390/nano12111802)
Supplement: Supplementary file 1 [file nanomaterials-12-01802-s001.zip › nanomaterials-1738318-supplementary.pdf]

Supplementary Materials

PdAg/Ag(111) Surface Alloys: A Highly Efficient Catalyst of Oxygen Reduction Reaction

Minghao Hua, Xuelei Tian *, Shuo Li and Xiaohang Lin *

Key Laboratory for Liquid-Solid Structural Evolution and Processing of Materials, Ministry of Education, School of Materials Science and Engineering, Shandong University, Jinan 250061, China;
huaminghao@outlook.com (M.H.); 18366111497@163.com (S.L.)
* Correspondence: lxh12345@sdu.edu.cn (X.L.); tianxuelei@sdu.edu.cn (X.T.)

Table S1 The free energies of the adsorption of ORR intermediates in eV on all active site models.

Active site model	ΔG_{*OOH}	ΔG_{*O}	ΔG_{OH*}
Pd_M@Ag(111)	4.15194	1.76072	0.78351
Pd_D@Ag(111)	4.08826	1.64927	0.80979
Pd_{T1}@Ag(111)	4.14847	1.62848	0.87055
Pd_{T2}@Ag(111)	4.16367	1.69931	0.85212
Pd_{T3}@Ag(111)	4.16664	1.67661	0.84570
Pd_M@Pd_{1L}Ag(111)	4.10261	1.84045	0.81721
Pd_D@Pd_{1L}Ag(111)	4.02477	1.63452	0.75966
Pd_{T1}@Pd_{1L}Ag(111)	3.98324	1.40795	0.78896
Pd_{T2}@Pd_{1L}Ag(111)	4.05015	1.54504	0.79159
Pd_{T3}@Pd_{1L}Ag(111)	4.04202	1.62215	0.75420
Pd_{1L}Ag(111)	4.23506	2.10597	0.82387

Table S2 *d*-band center and Bader charge of Pd atom at the first layer of all models

Active site model	*d*-band center	Bader charge
Pd_M@Ag(111)	−1.545	−0.229
Pd_D@Ag(111)	−1.496	−0.207
Pd_{T1}@Ag(111)	−1.617	−0.188
Pd_{T2}@Ag(111)	−1.609	−0.198
Pd_{T3}@Ag(111)	−1.593	−0.198
Pd_M@Pd_{1L}Ag(111)	−1.329	−0.154
Pd_D@Pd_{1L}Ag(111)	−1.292	−0.129
Pd_{T1}@Pd_{1L}Ag(111)	−1.426	−0.116
Pd_{T2}@Pd_{1L}Ag(111)	−1.410	−0.121
Pd_{T3}@Pd_{1L}Ag(111)	−1.394	−0.122

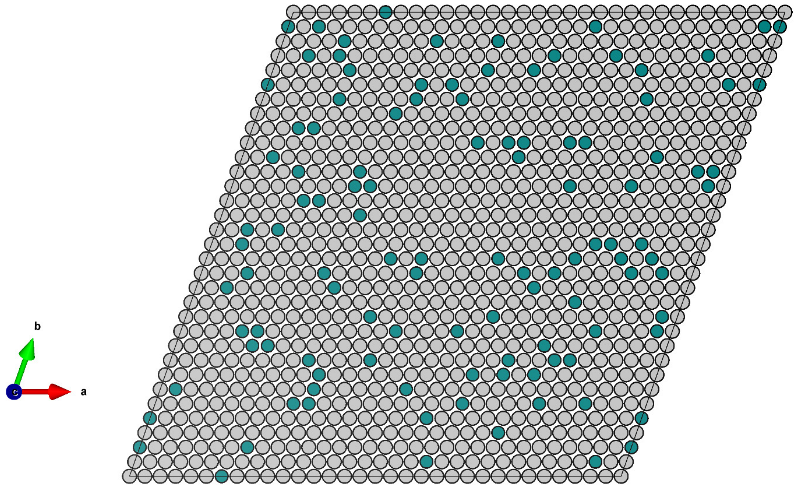

Figure S1 MC snapshots of the atomic structure of PdAg(111) with 50% Pd at 1200 K.[1] The silver and green balls represent Ag and Pd atoms, respectively.

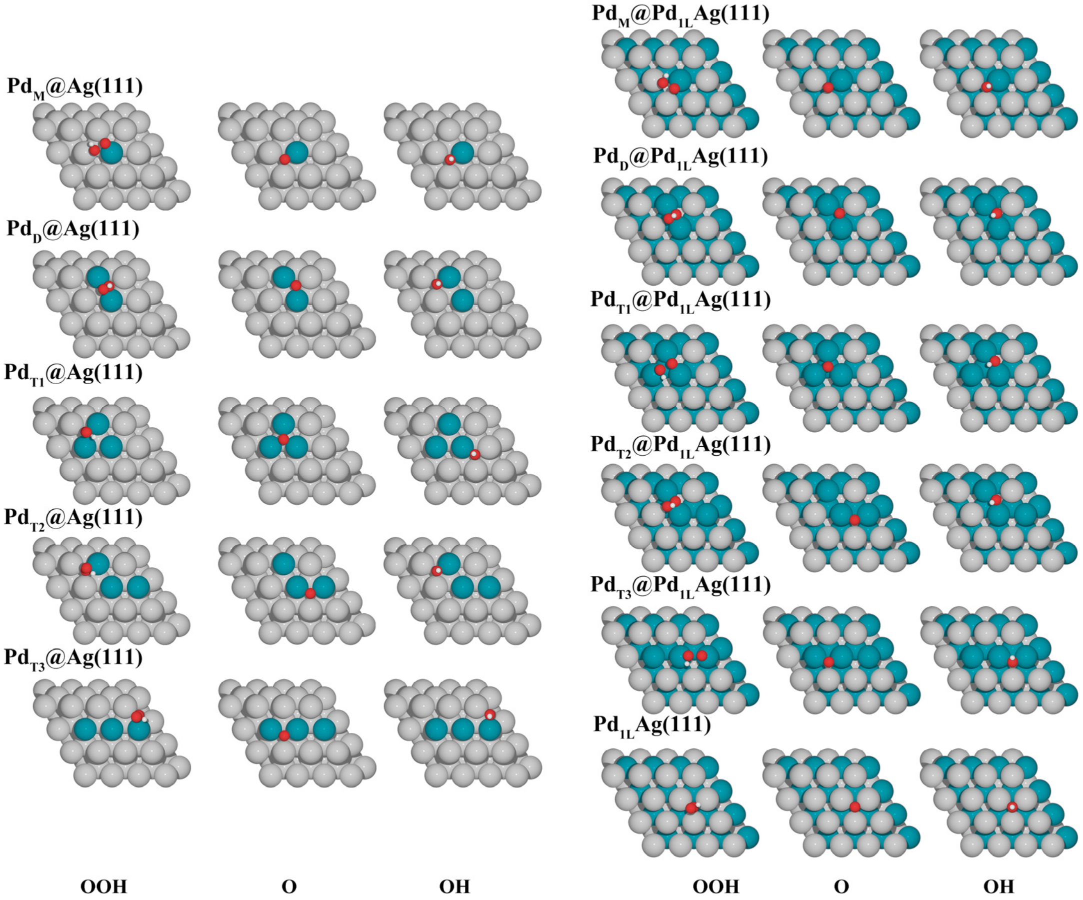

Figure S2 Top view of the atomistic structures of all ORR intermediates on all considered surfaces. The silver, cyan, red, and white balls represent Ag, Pd, O, and H atoms, respectively.

References

[1] Hua, M.; Tian, X.; Li, S.; Zhang, X.; Shao, A.; Song, L.; Lin, X. A casting combined quenching strategy to prepare PdAg single atom alloy designed by Cluster Expansion combined Monte Carlo method. *Phys. Chem. Chem. Phys.* 2021, doi:10.1039/d1cp05046j.
